# Supplementary material for: New Bisabolane-Type Sesquiterpenoids from Curcuma longa and Their Anti-Atherosclerotic Activity
Source: Molecules. 2023 Mar 16;28(6):2704. doi: 10.3390/molecules28062704 (PMC10058791; doi:10.3390/molecules28062704)
Supplement: Supplementary file 1 [file molecules-28-02704-s001.zip › Supporting Information.pdf]

Supporting Information

New bisabolane-type sesquiterpenoids from *Curcuma longa* and their anti-atherosclerotic activities

Yu-Qin Guo [1,2,3,†], Guang-Xu Wu [1,2,3†], Cheng Peng [1,2], Yun-Qiu Fan [1,2,3], Lei Li [1,2,3], Fei Liu [1,2,3,*], Liang Xiong [1,2,3,*]

1 State Key Laboratory of Southwestern Chinese Medicine Resources, Chengdu University of Traditional Chinese Medicine, Chengdu 611137, China; gyqthunder@163.com (Y.-Q.G.); wgx_julia@163.com (G.-X.W.)

2 School of Pharmacy, Chengdu University of Traditional Chinese Medicine, Chengdu 611137, China

3 Institute of Innovative Medicine Ingredients of Southwest Specialty Medicinal Materials, School of Pharmacy, Chengdu University of Traditional Chinese Medicine, Chengdu 611137, China

* Correspondence: liufei@cdutcm.edu.cn (F.L.); xiling@cdutcm.edu.cn, xiling0505@126.com (L.X.)

† These authors contributed equally to this work.

The List of Contents

No.	Content	Page
1	**Text S1.NMR Data Calculation of Compounds 1**	S1
2	**Figure S1.** *ω*B97XD/DGDZVP optimized 10 conformers of (6*S*,7*S*)-bisabol-2-en-11-ol-1,9-dione	S2
3	**Table S1.** Energy analysis for the conformers of (6*S*,7*S*)-bisabol-2-en-11-ol-1,9-dione	S2
4	**Figure S2.** *ω*B97XD/DGDZVP optimized eight conformers of (6*S*,7*R*)-bisabol-2-en-11-ol-1,9-dione	S3
5	**Table S2.** Energy analysis for the conformers of (6*S*,7*R*)-bisabol-2-en-11-ol-1,9-dione	S3
6	**Table S3.** Experimental ^{13}C NMR chemical shifts of compound **1** and GIAO isotropic magnetic shielding values calculated for the PCM/mPW1PW91/6-311+G (d,p) geometries of (6*S*,7*S*)/(6*R*,7*R*)-bisabol-2-en-11-ol-1,9-dione	S3
7	**Table S4.** Experimental ^{1}H-NMR chemical shifts of compound **1** and GIAO isotropic magnetic shielding values calculated for the PCM/mPW1PW91/6-311+G (d,p) geometries of (6*S*,7*S*)/(6*R*,7*R*)-bisabol-2-en-11-ol-1,9-dione	S4
8	**Table S5.** Experimental ^{13}C-NMR chemical shifts of compound **1** and GIAO isotropic magnetic shielding values calculated for PCM/mPW1PW91/6-311+G (d,p) geometries of (6*S*,7*R*)/(6*R*,7*S*)-bisabol-2-en-11-ol-1,9-dione	S5
9	**Table S6.** Experimental ^{1}H-NMR chemical shifts of compound **1** and GIAO isotropic magnetic shielding values calculated for PCM/mPW1PW91/6-311+G (d,p) geometries of (6*S*,7*R*)/(6*R*,7*S*)-bisabol-2-en-11-ol-1,9-dione.	S6
10	**Figure S3.** Regression analysis of the experimental ^{13}C NMR chemical shifts of compound **1** versus the calculated ^{13}C NMR chemical shifts of (6*S*,7*S*)/(6*R*,7*R*)-bisabol-2-en-11-ol-1,9-dione (A) and (6*S*,7*R*)/(6*R*,7*S*)-bisabol-2-en-11-ol-1,9-dione (B)	S7
11	**Figure S4.** The calculated shielding tensors, scaled shifts, and unscaled shifts for (6*S*,7*S*)/(6*R*,7*R*)-bisabol-2-en-11-ol-1,9-dione (isomer 1) and (6*S*,7*R*)/(6*R*,7*S*)-bisabol-2-en-11-ol-1,9-dione (isomer 2)	S7
12	**Table S7.** DP4+ probability (%) details of (6*S*,7*S*)/(6*R*,7*R*)-bisabol-2-en-11-ol-1,9-dione and (6*S*,7*R*)/(6*R*,7*S*)-bisabol-2-en-11-ol-1,9-dione for compound **1**	S8
13	**Figure S5.** DP4+ probability of (6*S*,7*S*)/(6*R*,7*R*)-bisabol-2-en-11-ol-1,9-dione and (6*S*,7*R*)/(6*R*,7*S*)-bisabol-2-en-11-ol-1,9-dione for compound **1**	S8
14	**Text S2. NMR Data Calculation of Compound 2**	S8
15	**Table S8.** Experimental ^{13}C NMR chemical shifts of compound **2** and GIAO isotropic magnetic shielding values calculated for the PCM/mPW1PW91/6-311+G (d,p) geometries of (6*S*,7*S*)/(6*R*,7*R*)-bisabol-2-en-11-ol-1,9-dione	S9

16 **Table S9.** Experimental ^{1}H-NMR chemical shifts of compound **2** and GIAO isotropic magnetic shielding values calculated for the PCM/mPW1PW91/6-311+G (d,p) geometries of (6*S*,7*S*)/(6*R*,7*R*)-bisabol-2-en-11-ol-1,9-dione S10

17 **TableS10.** Experimental ^{13}C-NMR chemical shifts of compound **2** and GIAO isotropic magnetic shielding values calculated for PCM/mPW1PW91/6-311+G (d,p) geometries of (6*S*,7*R*)/(6*R*,7*S*)-bisabol-2-en-11-ol-1,9-dione S11

18 **Table S11.** Experimental ^{1}H-NMR chemical shifts of compound **2** and GIAO isotropic magnetic shielding values calculated for PCM/mPW1PW91/6-311+G (d,p) geometries of (6*S*,7*R*)/(6*R*,7*S*)-bisabol-2-en-11-ol-1,9-dione S11

19 **Figure S6.** Regression analysis of the experimental ^{13}C NMR chemical shifts of compound **2** versus the calculated ^{13}C NMR chemical shifts of (6*S*,7*S*)/(6*R*,7*R*)-bisabol-2-en-11-ol-1,9-dione (A) and (6*S*,7*R*)/(6*R*,7*S*)-bisabol-2-en-11-ol-1,9-dione (B) S12

20 **Figure S7.** The calculated shielding tensors, scaled shifts, and unscaled shifts for (6*S*,7*S*)/(6*R*,7*R*)-bisabol-2-en-11-ol-1,9-dione (isomer 1) and (6*S*,7*R*)/(6*R*,7*S*)-bisabol-2-en-11-ol-1,9-dione (isomer 2) S13

21 **Table S12.** DP4+ probability (%) details of (6*S*,7*S*)/(6*R*,7*R*)-bisabol-2-en-11-ol-1,9-dione and (6*S*,7*R*)/(6*R*,7*S*)-bisabol-2-en-11-ol-1,9-dione for compound **2** S13

22 **Figure S8.** DP4+ probability of (6*S*,7*S*)/(6*R*,7*R*)-bisabol-2-en-11-ol-1,9-dione and (6*S*,7*R*)/(6*R*,7*S*)-bisabol-2-en-11-ol-1,9-dione for compound **2** S14

23 **Text S3. ECD Calculation of Compound 1** S14

24 **Figure S9.** Experimental and calculated ECD spectra of compound **1** S15

25 **Text S4. ECD Calculation of Compound 2** S15

26 **Figure S10.** Experimental and calculated ECD spectra of compound **2** S16

27 **Text S5. ECD Calculation of Compound 3** S16

28 **Figure S11.** *ω*B97XD/DGDZVP optimized seven conformers of (6*S*,7*S*)-bisabol-2,10-dien-1,9-dione S17

29 **Table S13.** Energy analysis for the conformers of (6*S*,7*S*)-bisabol-2,10-dien-1,9-dione S17

30 **Figure S12.** *ω*B97XD/DGDZVP optimized 15 conformers of (6*S*,7*R*)-bisabol-2,10-dien-1,9-dione S18

31 **Table S14.** Energy analysis for the conformers of (6*S*,7*R*)-bisabol-2,10-dien-1,9-dione S18

32 **Figure S13.** Experimental and calculated ECD spectra of compound **3** S19

33 **Text S6. ECD Calculation of Compound 4** S19

34 **Figure S14.** Experimental and calculated ECD spectra of compound **4** S20

35 **7. Original Spectra of New Compounds** S21

36 **Figure S15**. The UV spectrum of compound **1** in MeCN S21

37 **Figure S16**. The IR spectrum of compound **1** S22

Figure S17. The (+)-HRESIMS spectroscopic data of compound **1** S23
Figure S18. The ^{1}H NMR spectrum of compound **1** in acetone-d_6 S24
Figure S19. The ^{13}C NMR spectrum of compound **1** in acetone-d_6 S25
Figure S20. The DEPT spectrum of compound **1** in acetone-d_6 S26
Figure S21. The HSQC spectrum of compound **1** in acetone-d_6 S27
Figure S22. The ^{1}H-^{1}H COSY spectrum of compound **1** in acetone-d_6 S28
Figure S23. The HMBC spectrum of compound **1** in acetone-d_6 S29
Figure S24. The UV spectrum of compound **2** in MeCN S30
Figure S25. The IR spectrum of compound **2** S31
Figure S26. The (+)-HRESIMS spectroscopic data of compound **2** S32
Figure S27. The ^{1}H NMR spectrum of compound **2** in acetone-d_6 S33
Figure S28. The ^{13}C NMR spectrum of compound **2** in acetone-d_6 S34
Figure S29. The DEPT spectrum of compound **2** in acetone-d_6 S35
Figure S30. The HSQC spectrum of compound **2** in acetone-d_6 S36
Figure S31. The ^{1}H-^{1}H COSY spectrum of compound **2** in acetone-d_6 S37
Figure S32. The HMBC spectrum of compound **2** in acetone-d_6 S38
Figure S33. The UV spectrum of compound **3** in MeCN S39
Figure S34. The IR spectrum of compound **3** S40
Figure S35. The (+)-HRESIMS spectroscopic data of compound **3** S41
Figure S36. The ^{1}H NMR spectrum of compound **3** in $CDCl_3$ S42
Figure S37. The ^{13}C NMR spectrum of compound **3** in $CDCl_3$ S43
Figure S38. The DEPT spectrum of compound **3** in $CDCl_3$ S44
Figure S39. The HSQC spectrum of compound **3** in $CDCl_3$ S45
Figure S40. The ^{1}H-^{1}H COSY spectrum of compound **3** in $CDCl_3$ S46
Figure S41. The HMBC spectrum of compound **3** in $CDCl_3$ S47
Figure S42. The UV spectrum of compound **4** S48
Figure S43. The IR spectrum of compound **4** S49
Figure S44. The (+)-HRESIMS spectroscopic data of compound **4** S50
Figure S45. The ^{1}H NMR spectrum of compound **4** in $CDCl_3$ S51
Figure S46. The ^{13}C NMR spectrum of compound **4** in $CDCl_3$ S52
Figure S47. The DEPT spectrum of compound **4** in $CDCl_3$ S53

Figure S48. The HSQC spectrum of compound **4** in $CDCl_3$ S54

Figure S49. The ^1H-^1H COSY spectrum of compound **4** in $CDCl_3$ S55

Figure S50. The HMBC spectrum of compound **4** in $CDCl_3$ S56

Text S1. NMR Data Calculation of Compound 1.

Conformation searches based on molecular mechanics with MMFF94s force field were performed for (6*S*,7*S*)-bisabol-2-en-11-ol-1,9-dione and (6*S*,7*R*)-bisabol-2-en-11-ol-1,9-dione and gave 10 and eight conformers having relative energy within 3.5 kcal/mol, respectively [1]. The conformers were optimized using DFT at the B3LYP/6–31G (d) level in vacuum with the Gaussian 16 program (Tables S1 and S2) [2]. The B3LYP/6–31G (d)-optimized conformers were then reoptimized at the *ω*B97XD/DGDZVP level in acetone. NMR chemical shift calculation for the *ω*B97XD/DGDZVP-optimized 10 conformers of (6*S*,7*S*)-bisabol-2-en-11-ol-1,9-dione and eight conformers of (6*S*,7*R*)-bisabol-2-en-11-ol-1,9-dione (Boltzmann distribution ≥ 1%, Figures S1 and S2) were carried out at the PCM/mPW1PW91/6–311+G (d,p) level in acetone with GIAO method [3]. The calculated ^{13}C- and ^{1}H-NMR data of these conformers were averaged according to the Boltzmann distribution theory and their relative Gibbs free energy. Tables S3 and S4 show the calculated ^{13}C- and ^{1}H-NMR data of (6*S*,7*S*)/(6*R*,7*R*)-bisabol-2-en-11-ol-1,9-dione. Tables S5 and S6 show the calculated ^{13}C- and ^{1}H-NMR data of (6*S*,7*R*)/(6*R*,7*S*)-bisabol-2-en-11-ol-1,9-dione. Linear correlation coefficients (R^2) were calculated for the comparative evaluation of the experimental data of compound **1** and the calculated data of (6*S*,7*S*)/(6*R*,7*R*)-bisabol-2-en-11-ol-1,9-dione and (6*S*,7*R*)/(6*R*,7*S*)-

bisabol-2-en-11-ol-1,9-dione, respectively (Figure S3). The isotropic values of TMS were calculated in the same level and used as references. The DP4+ parameters were calculated using the excel file, which was provided by Ariel M. Sarotti (Figure S4 and Table S7) [4]. The DP4+ probability shows that (6*S*,7*S*)/(6*R*,7*R*)-bisabol-2-en-11-ol-1,9-dione is the preponderant isomer of compound **1** (Figure S5), in contrast to (6*S*,7*R*)/(6*R*,7*S*)-bisabol-2-en-11-ol-1,9-dione.

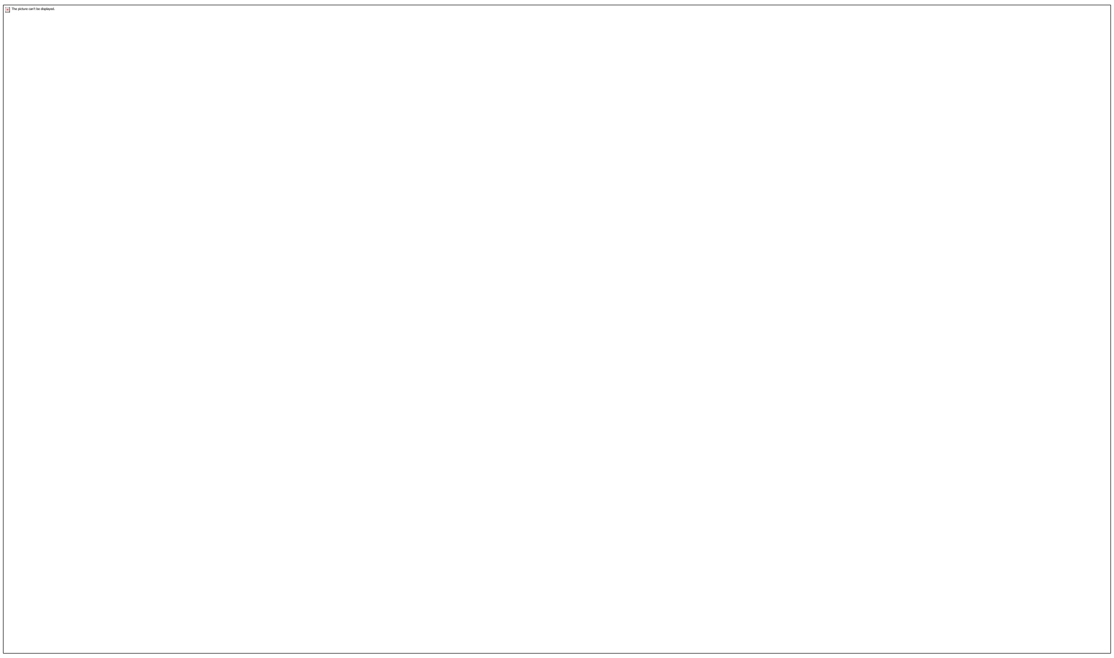

Figure S1. *ω*B97XD/DGDZVP optimized 10 conformers of (6*S*,7*S*)-bisabol-2-en-11-ol-1,9-dione.

Table S1. Energy analysis for the conformers of (6*S*,7*S*)-bisabol-2-en-11-ol-1,9-dione.

Conf.	MMFF energy	BL3YP/6-31G(d) Gibbs free energy (298.15 K)			*ω*B97XD/DGDZVP Gibbs free energy (298.15 K)		
	ΔE (Kcal/mol)	G (Hartree)	ΔG (Kcal/mol)	Boltzmann distribution	G (Hartree)	ΔG (Kcal/mol)	Boltzmann distribution
C1	0.0000	-811.405834	0.0000	0.018	-811.257818	0.0000	0.029
C2	0.0512	-811.404704	0.7090	0.005	-811.259006	-0.7450	0.104
C3	0.3523	-811.40551	0.2030	0.012	-811.257992	-0.1090	0.035

C4	1.0070	-811.408706	-1.8020	0.368	-811.258147	-0.2060	0.042
C5	1.2731	-811.407123	-0.8090	0.069	-811.259668	-1.1610	0.209
C6	1.3122	-811.405526	0.1930	0.013	-811.258107	-0.1810	0.04
C7	1.3835	-811.40422	1.0130	0.003	-811.255792	1.2710	0.003
C8	1.5083	-811.406969	-0.7120	0.058	-811.260162	-1.4710	0.352
C9	1.6253	-811.404139	1.0640	0.003	-811.257392	0.2670	0.019
C10	1.6795	-811.408899	-1.9230	0.451	-811.259456	-1.0280	0.167

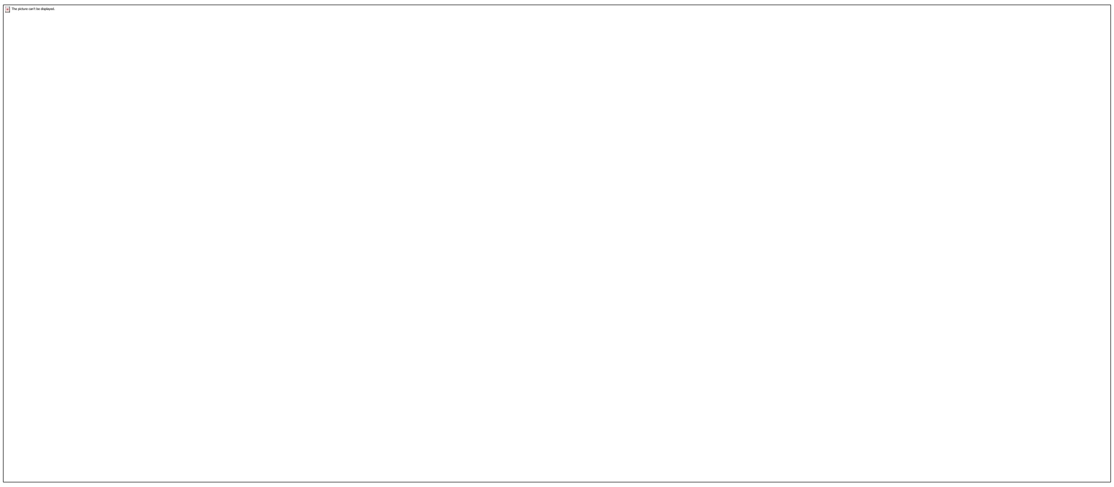

Figure S2. ωB97XD/DGDZVP optimized eight conformers of (6*S*,7*R*)-bisabol-2-en-11-ol-1,9-dione.

Table S2. Energy analysis for the conformers of (6*S*,7*R*)-bisabol-2-en-11-ol-1,9-dione.

Conf.	MMFF energy	BL3YP/6-31G(d) Gibbs free energy (298.15 K)			ωB97XD/DGDZVP Gibbs free energy (298.15 K)		
	ΔE (Kcal/mol)	G (Hartree)	ΔG (Kcal/mol)	Boltzmann distribution	G (Hartree)	ΔG (Kcal/mol)	Boltzmann distribution
C1	0.0000	-811.404816	0.0000	0.137	-811.257705	0.0000	0.135
C2	0.0767	-811.405523	-0.4440	0.29	-811.258829	-0.7050	0.446
C3	0.9565	-811.404192	0.3920	0.071	-811.254783	1.8340	0.006
C4	1.0601	-811.404672	0.0900	0.118	-811.255977	1.0840	0.022
C5	1.2836	-811.40271	1.3220	0.015	-811.25552	1.3710	0.013
C6	1.4915	-811.405302	-0.3050	0.23	-811.258442	-0.4620	0.296
C7	1.7246	-811.403978	0.5260	0.056	-811.257194	0.3210	0.079
C8	2.0654	-811.404342	0.2970	0.083	-811.254253	2.1660	0.003

Table S3. Experimental ^{13}C NMR chemical shifts of compound **1** and GIAO isotropic magnetic shielding values calculated for the PCM/mPW1PW91/6–311+G (d,p) geometries of (6*S*,7*S*)/(6*R*,7*R*)-bisabol-2-en-11-ol-1,9-dione.

NO.	Expt-1	C1	C2	C3	C4	C5	C6	C7	C8	C9	C10	Averaged[a]	Unscaled shifts (δ_U)[b]	Scaled shifts (δ_S)[c]
**1**	127.3	54.3887	55.5904	55.6306	54.7917	55.6643	56.4857	55.9955	55.7939	54.732	54.6761	55.4834924	133.375308	123.322046
**2**	161.7	6.5998	6.5904	6.5687	11.0249	9.4442	9.5502	7.8027	9.5088	6.6975	10.8584	8.0688642	180.789936	167.098824
**3**	31.5	153.1686	153.1657	153.491	152.9131	153.5127	153.635	152.9618	153.2439	155.4268	153.2236	153.221341	35.637460	33.083057
**4**	25.2	157.0092	162.7781	162.0975	156.0488	163.0273	164.8121	163.5629	162.926	157.0221	155.9219	161.619658	27.239142	25.329095
**5**	51	139.9022	132.4493	132.7498	139.3156	133.1282	134.5121	134.6808	133.7658	140.6166	139.6148	134.065387	54.793413	50.769285
**6**	198.6	-26.6384	-25.1549	-26.7829	-23.2094	-21.3966	-19.7587	-23.3892	-21.442	-25.6075	-23.157	-23.864818	212.723618	196.582419
**7**	55.5	127.3422	132.3769	127.8302	133.3621	135.1428	133.575	126.734	135.539	127.6349	132.7057	133.076937	55.781863	51.681897
**8**	211.9	-39.2851	-39.3818	-39.8265	-43.7059	-44.3127	-42.3009	-41.6233	-44.5258	-38.5869	-41.629	-41.243877	230.102677	212.628083
**9**	49	134.3231	135.3741	138.2592	132.5793	136.6412	137.2653	138.7731	134.8804	134.0623	134.1981	135.127075	53.731725	49.789054
**10**	28.3	152.3595	155.4751	157.6822	153.194	158.1833	154.5629	158.11	156.8202	149.5026	153.4809	155.508895	33.349905	30.971013
**11**	69.9	113.8242	113.9769	113.5491	113.1207	113.8975	115.4422	113.3391	114.4161	113.6014	114.5437	114.007486	74.851314	69.288259
**12**	29.8	159.2303	153.9603	159.2028	156.8912	157.2473	159.5876	155.3128	160.4259	158.7847	160.4468	156.256802	32.601998	30.280489
**13**	29.8	155.3653	160.8389	155.6822	159.451	159.5207	157.3406	160.4165	157.3768	155.0855	157.446	159.570083	29.288717	27.221417
**14**	17.4	173.1396	169.727	167.9631	173.8594	170.4103	165.759	165.2485	170.2899	170.2685	173.8222	170.499474	18.359326	17.130575
**15**	24	161.1813	161.1261	161.0415	161.5986	161.236	161.0932	161.0424	161.3521	160.7548	161.535	161.239301	27.619499	25.680268

[a] Averaged according to the Boltzmann-calculated contribution at the B3LYP/6-31+G (d,p) level.

[b] δ_U = Calculated Shielding Value (TMS) – Calculated Shielding Value (Averaged).

[c] $\delta_S = (\delta_U + 0.1948)/1.0831$.

Table S4. Experimental ^{1}H-NMR chemical shifts of compound **1** and GIAO isotropic magnetic shielding values calculated for the PCM/mPW1PW91/6–311+G (d,p) geometries of (6*S*,7*S*)/(6*R*,7*R*)-bisabol-2-en-11-ol-1,9-dione.

NO.	Expt-1	C1	C2	C3	C4	C5	C6	C7	C8	C9	C10	Averaged[a]
**1**	5.74	25.6731	25.5355	25.6123	25.8051	25.5552	25.774	25.6288	25.6041	25.5773	25.7733	25.5972348
**3a**	2.37	29.3634	29.3462	29.341	29.3192	29.3797	29.4678	29.3816	29.3839	29.5225	29.3422	29.3560408
**3b**	2.37	29.485	29.4231	29.4365	29.5092	29.4657	29.4485	29.3495	29.4661	29.2008	29.5248	29.4483945
**4a**	1.74	30.1149	29.8491	29.7992	30.0806	29.867	30.0097	29.9656	29.8968	29.6398	30.0721	29.8995152
**4b**	2	29.7847	30.3119	30.2952	29.8239	30.3764	29.5609	29.375	30.3531	29.8418	29.8339	30.2195544
**5**	2.17	29.0791	29.4844	29.5747	29.2388	29.5359	29.8295	29.7267	29.5844	29.4882	29.5356	29.4866166
**7a**	2.6	29.6123	28.2174	29.6601	29.6999	29.5993	28.4835	28.6912	28.6131	29.602	28.956	28.6555603
**7b**	2.6	28.8951	29.7156	28.9951	28.9636	28.1227	29.4029	29.7211	29.3494	28.9313	29.5164	29.3905212
**9a**	2.37	27.7475	28.9356	29.0458	27.5938	29.5394	29.7822	29.7636	29.4929	28.4562	27.8257	28.8713101
**9b**	2.51	29.3958	30.0853	30.0106	29.2676	30.1371	29.4047	29.1752	29.9727	29.6332	29.4738	29.9264784
**10**	2.64	29.8783	28.9062	29.2068	29.8966	28.9936	28.525	28.6901	28.767	29.4828	29.8279	29.0656003
**12**	1.2	30.8238	30.473	30.5161	30.628	30.5953	30.8852	30.5993	30.7344	30.5764	30.7365	30.5706206
**12**	1.2	31.3303	30.4157	30.4668	30.6671	30.6165	30.4835	30.5293	30.4919	30.784	30.4278	30.4887882
**12**	1.2	30.761	30.569	30.5896	30.7332	30.6559	30.6408	30.7885	30.8685	30.6174	30.8955	30.6651565
**13**	1.2	30.6279	30.7173	30.594	30.8058	30.6508	30.7248	30.8038	30.6236	30.5592	30.682	30.6988198
**13**	1.2	30.4452	30.9365	30.5012	31.1913	30.5735	30.8044	30.1362	30.6293	30.4264	30.725	30.8452205
**13**	1.2	30.8294	30.8554	30.7755	30.7991	30.6577	30.8072	30.9044	30.7272	30.7644	30.851	30.8085834
**14**	0.91	30.6621	31.0896	31.0167	30.8313	31.1118	30.8753	30.9432	31.0558	30.6407	30.8612	31.0319349
**14**	0.91	31.1816	30.8597	30.2908	31.2328	30.741	30.756	30.7971	30.944	31.0672	31.2453	30.9190909
**14**	0.91	31.0565	30.9103	30.9631	30.6957	30.9027	30.7159	30.7285	30.9002	31.2264	30.7174	30.8761825
**15**	1.93	29.8246	29.8156	29.8215	29.8784	29.816	29.8505	29.7805	29.8345	29.7751	29.8734	29.828866
**15**	1.93	29.692	29.6777	29.6667	29.7663	29.733	29.685	29.5919	29.7365	29.7984	29.7359	29.7052643
**15**	1.93	25.6731	25.5355	25.6123	25.8051	25.5552	25.774	25.6288	25.6041	25.5773	25.7733	29.7948757

[a] Averaged according to the Boltzmann-calculated contribution at the B3LYP/6-31+G (d,p) level.

Table S5. Experimental ^{13}C-NMR chemical shifts of compound **1** and GIAO isotropic magnetic shielding values calculated for PCM/mPW1PW91/6–311+G (d,p) geometries of (6*S*,7*R*)/(6*R*,7*S*)-bisabol-2-en-11-ol-1,9-dione.

NO	Expt-1	C1	C2	C3	C4	C5	C6	C7	C8	Averaged[a]	Unscaled shifts (δ_U)[b]	Scaled shifts (δ_S)[c]
**1**	127.3	57.3649	56.3223	55.3283	55.9175	55.7725	56.375	57.0258	55.3046	56.4451763	132.4136237	122.7626709
**2**	161.7	6.793	8.7647	7.166	7.4958	7.9054	10.9984	10.0705	11.0427	9.371124	179.487676	166.5443415
**3**	31.5	158.0289	157.9436	153.4627	152.9955	152.7507	157.9903	158.3623	153.1142	157.647426	31.2113736	28.63855432
**4**	25.2	159.9795	159.2796	157.6416	158.0158	157.8027	160.019	160.6429	156.7999	159.517534	29.3412659	26.89924284
**5**	51	139.7507	132.2417	139.7688	134.8381	133.9449	132.1158	138.4577	138.7231	133.568907	55.2898926	51.03301023
**6**	198.6	-27.9846	-27.7856	-26.8938	-26.4957	-26.2177	-25.4816	-25.8186	-23.0533	-26.742434	215.6012341	200.1321002
**7**	55.5	128.1135	129.9107	127.4123	128.1899	132.7298	132.4755	131.6449	133.6223	130.598375	58.2604252	53.79578237
**8**	211.9	-37.8394	-35.3133	-38.9939	-38.8589	-39.1688	-38.6652	-41.4229	-40.8296	-37.390045	226.2488448	210.0350119
**9**	49	137.1837	131.5333	135.0556	131.9401	129.7951	136.1903	139.8898	138.7383	134.433951	54.424849	50.22846819
**10**	28.3	154.5545	158.7769	153.1208	157.3721	153.8673	156.1269	154.7858	152.9894	156.691724	32.1670764	29.52741481
**11**	69.9	113.6082	113.9332	113.8921	113.4789	113.8749	115.2836	115.1914	114.7211	114.350794	74.5080061	68.90699972
**12**	29.8	155.5366	160.4241	155.5378	159.2323	153.7953	157.5049	159.6427	159.6492	158.343653	30.5151474	27.99102251
**13**	29.8	159.7437	155.3684	159.5449	155.6733	160.6707	159.2074	157.3302	157.24	157.447467	31.4113332	28.82452865
**14**	17.4	168.0626	166.5024	166.2217	167.6177	170.0482	166.9302	167.1703	165.1678	166.801528	22.0572725	20.12469541
**15**	24	161.138	161.1711	161.3803	161.432	161.4883	161.3015	161.3085	161.7409	161.07391	27.78489	122.7626709

[a] Averaged according to the Boltzmann-calculated contribution at B3LYP/6–31+G (d,p) level.

[b] δ_U = Calculated Shielding Value (TMS) – Calculated Shielding Value (Averaged).

[c] $\delta_S = (\delta_U - 0.4192)/1.0752$.

Table S6. Experimental ^{1}H-NMR chemical shifts of compound **1** and GIAO isotropic magnetic shielding values calculated for PCM/mPW1PW91/6–311+G (d,p) geometries of (6*S*,7*R*)/(6*R*,7*S*)-bisabol-2-en-11-ol-1,9-dione.

NO	Expt-1	C1	C2	C3	C4	C5	C6	C7	C8	Averaged[a]
**1**	5.74	25.7618	25.7836	25.7271	25.6921	25.6044	25.9098	25.7853	25.8192	25.7982767
**3a**	2.37	29.6846	29.6854	29.362	29.3461	29.317	29.7102	29.7117	29.3573	29.6530775
**3b**	2.37	29.3581	29.2505	29.484	29.468	29.4386	29.2702	29.3792	29.51	29.2591376
**4a**	1.74	29.8812	29.9463	29.6267	29.7406	29.7301	29.969	29.9092	29.5683	29.904779
**4b**	2	29.6933	29.7234	30.3437	30.3681	30.2781	29.7327	29.7291	30.3556	29.7174904
**5**	2.17	29.1543	30.1833	28.87	29.7458	29.6956	30.1715	29.4841	29.1869	29.9451962
**7a**	2.6	29.7212	29.0433	29.6847	28.9515	29.7522	28.9792	29.2699	29.3122	29.1045506
**7b**	2.6	29.0321	29.8126	28.9436	29.5999	28.1286	29.3238	29.0171	28.6368	29.4168118
**9a**	2.37	28.7781	29.5184	27.252	29.7524	30.0377	29.8786	29.3371	28.0057	29.5082059
**9b**	2.51	29.454	28.5753	29.4413	28.5315	28.5669	29.0339	29.7115	29.6645	28.911281
**10**	2.64	30.0809	29.3178	30.2113	29.8163	29.7228	29.0951	29.9799	30.4155	29.3688315

**12**	1.2	30.5707	30.7499	30.6203	30.5277	30.4308	30.7045	30.6956	30.8697	30.6664871
**12**	1.2	30.519	30.2138	30.4663	30.4644	30.407	30.8016	29.949	30.5248	30.4377242
**12**	1.2	30.7836	30.8627	30.833	30.5816	30.5587	30.8291	30.6217	30.9034	30.7849532
**13**	1.2	30.5883	30.5661	30.8394	30.5515	30.6645	30.786	30.6603	30.6921	30.6293774
**13**	1.2	30.3522	30.5607	31.2229	30.483	30.7776	30.2398	30.7803	30.7584	30.4006674
**13**	1.2	30.6388	30.7759	30.7841	30.7493	30.8236	30.7538	30.8067	30.8009	30.720573
**14**	0.91	30.5594	30.4456	30.6293	30.8373	30.9489	30.3723	30.4547	30.4956	30.4179299
**14**	0.91	30.9111	31.0146	31.121	30.7466	31.0354	30.9729	30.7776	31.0012	30.9352058
**14**	0.91	30.0273	31.021	30.3423	31.2766	31.2847	31.1163	30.0599	30.2731	30.837372
**15**	1.93	29.8124	29.741	29.8266	29.8262	29.7952	29.8259	29.8415	29.8709	29.7617968
**15**	1.93	29.7899	29.7093	29.6924	29.6867	29.6785	29.7399	29.8262	29.7422	29.70809
**15**	1.93	29.6787	29.6483	29.7672	29.7945	29.7988	29.6923	29.7044	29.8274	29.6482681

[a] Averaged according to the Boltzmann-calculated contribution at the B3LYP/6–31+G (d,p) level.

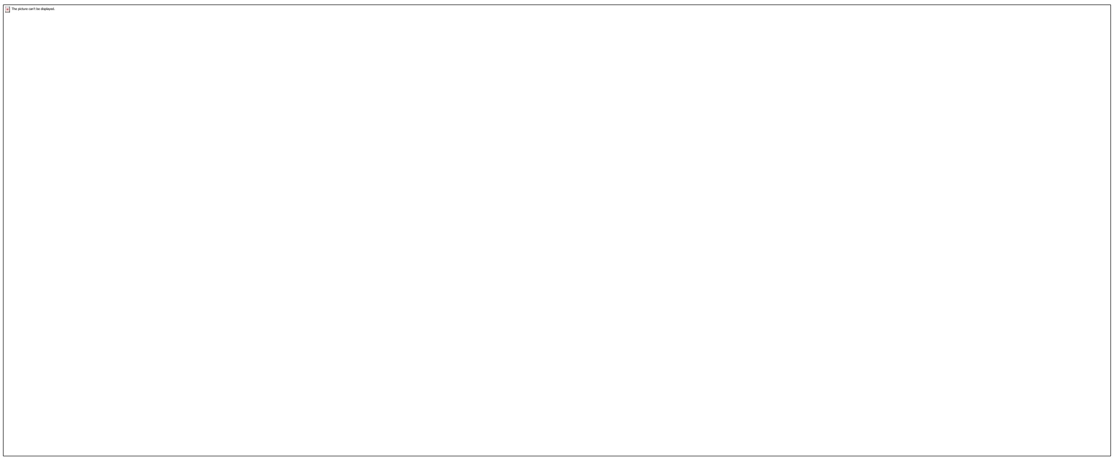

Figure S3. Regression analysis of the experimental ^{13}C NMR chemical shifts of compound **1** versus the calculated ^{13}C NMR chemical shifts of (6*S*,7*S*)/(6*R*,7*R*)-bisabol-2-en-11-ol-1,9-dione (A) and (6*S*,7*R*)/(6*R*,7*S*)-bisabol-2-en-11-ol-1,9-dione (B).

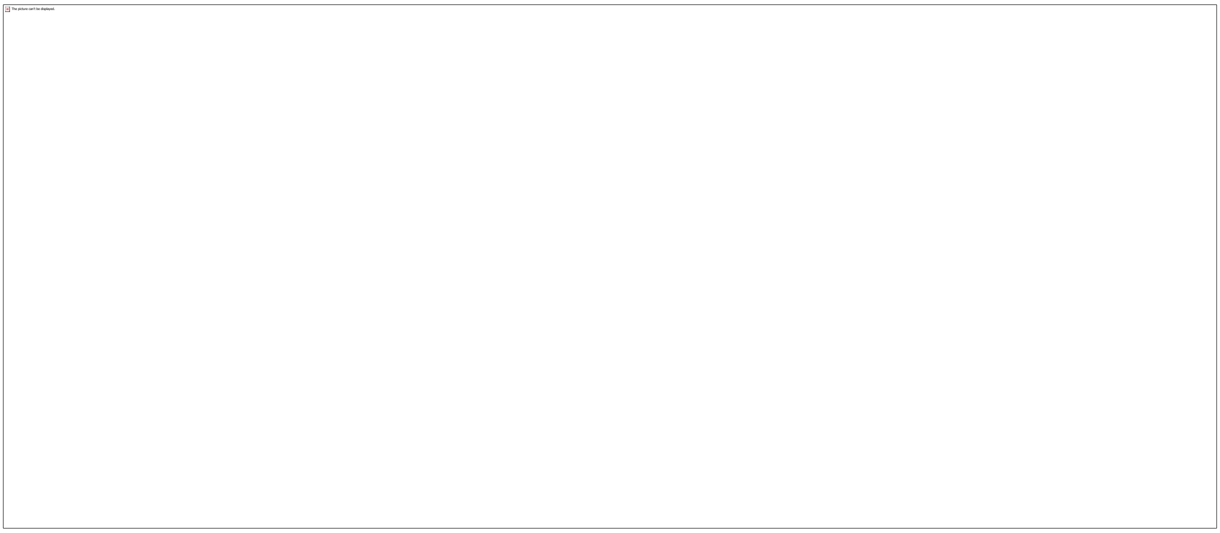

Figure S4. The calculated shielding tensors, scaled shifts, and unscaled shifts for (6*S*,7*S*)/(6*R*,7*R*)-bisabol-2-en-11-ol-1,9-dione (isomer 1) and (6*S*,7*R*)/(6*R*,7*S*)-bisabol-2-en-11-ol-1,9-dione (isomer 2).

Table S7. DP4+ probability (%) details of (6*S*,7*S*)/(6*R*,7*R*)-bisabol-2-en-11-ol-1,9-dione and (6*S*,7*R*)/(6*R*,7*S*)-bisabol-2-en-11-ol-1,9-dione for compound **1**.

		**Level of Theory: mPW1PW91/6–311+G (d,p) (acetone-*d*$_6$, PCM)**	
		(6*S*,7*S*)/(6*R*,7*R*)-bisabol-2-en-11-ol-1,9-dione	(6*S*,7*R*)/(6*R*,7*S*)-bisabol-2-en-11-ol-1,9-dione
sDP4+	^{1}*H*-date	54.22	45.78
	^{13}*C*-date	80.75	19.25
	All-date	83.25	16.75
uDP4+	^{1}*H*-date	50.13	49.87
	^{13}*C*-date	1.25	98.75
	All-date	1.26	98.74

DP4+	^{1}*H*-date	99.12	0.88
	^{13}*C*-date	4.73	95.27
	All-date	84.84	15.16

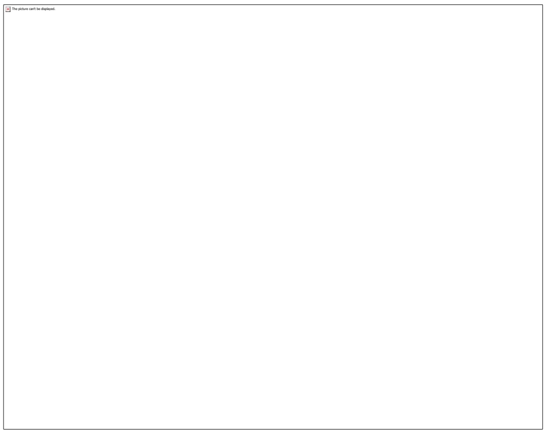

Figure S5. DP4+ probability of (6*S*,7*S*)/(6*R*,7*R*)-bisabol-2-en-11-ol-1,9-dione and (6*S*,7*R*)/(6*R*,7*S*)-bisabol-2-en-11-ol-1,9-dione for compound **1**.

Text S2. NMR Data Calculation of Compound 2.

The NMR chemical shift calculations for (6*S*,7*S*)/(6*R*,7*R*)-bisabol-2-en-11-ol-1,9-dione and (6*S*,7*R*)/(6*R*,7*S*)-bisabol-2-en-11-ol-1,9-dione are the same as described in section 1. The calculated ^{13}C- and ^{1}H-NMR data of (6*S*,7*S*)/(6*R*,7*R*)-bisabol-2-en-11-ol-1,9-dione and (6*S*,7*R*)/(6*R*,7*S*)-bisabol-2-en-11-ol-1,9-dione and the experimental ^{13}C- and ^{1}H-NMR data of compound **2** are shown in Tables S8–S11. Linear correlation coefficients (R^2) were calculated for the comparative evaluation of the experimental data of compound **2** and the calculated data of (6*S*,7*S*)/(6*R*,7*R*)-bisabol-2-en-11-ol-1,9-dione and (6*S*,7*R*)/(6*R*,7*S*)-bisabol-2-en-11-ol-1,9-dione, respectively (Figure S6). The isotropic values of TMS were calculated in the same level and used as references. The DP4+ parameters were calculated using the excel file, which was

provided by Ariel M. Sarotti (Figure S7 and Table S12).[4] The DP4+ probability shows that (6*S*,7*R*)/(6*R*,7*S*)-bisabol-2-en-11-ol-1,9-dione is the preponderant isomer of compound **2** (Figure S8), in contrast to (6*S*,7*S*)/(6*R*,7*R*)-bisabol-2-en-11-ol-1,9-dione.

Table S8. Experimental ^{13}C NMR chemical shifts of compound **2** and GIAO isotropic magnetic shielding values calculated for the PCM/mPW1PW91/6–311+G (d,p) geometries of (6*S*,7*S*)/(6*R*,7*R*)-bisabol-2-en-11-ol-1,9-dione.

NO.	Expt-2	C1	C2	C3	C4	C5	C6	C7	C8	C9	C10	Averaged[a]	Unscaled shifts (δ_U)[b]	Scaled shifts (δ_S)[c]
1	127.2	54.3887	55.5904	55.6306	54.7917	55.6643	56.4857	55.9955	55.7939	54.732	54.6761	55.4834924	133.37531	119.625135
2	162.4	6.5998	6.5904	6.5687	11.0249	9.4442	9.5502	7.8027	9.5088	6.6975	10.8584	8.0688642	180.78994	163.653947
3	31.1	153.1686	153.1657	153.491	152.9131	153.5127	153.635	152.9618	153.2439	155.4268	153.2236	153.221341	35.63746	28.866617
4	23.8	157.0092	162.7781	162.0975	156.0488	163.0273	164.8121	163.5629	162.926	157.0221	155.9219	161.619658	27.23914	21.068012
5	50.2	139.9022	132.4493	132.7498	139.3156	133.1282	134.5121	134.6808	133.7658	140.6166	139.6148	134.065387	54.79341	46.654669
6	199.9	-26.6384	-25.1549	-26.7829	-23.2094	-21.3966	-19.7587	-23.3892	-21.442	-25.6075	-23.157	-23.864818	212.72362	193.307288
7	55	127.3422	132.3769	127.8302	133.3621	135.1428	133.575	126.734	135.539	127.6349	132.7057	133.076937	55.78186	47.572535
8	211.5	-39.2851	-39.3818	-39.8265	-43.7059	-44.3127	-42.3009	-41.6233	-44.5258	-38.5869	-41.629	-41.243877	230.10268	209.445331
9	49.8	134.3231	135.3741	138.2592	132.5793	136.6412	137.2653	138.7731	134.8804	134.0623	134.1981	135.127075	53.73172	45.668794
10	27.3	152.3595	155.4751	157.6822	153.194	158.1833	154.5629	158.11	156.8202	149.5026	153.4809	155.508895	33.34990	26.742413
11	69.9	113.8242	113.9769	113.5491	113.1207	113.8975	115.4422	113.3391	114.4161	113.6014	114.5437	114.007486	74.85131	65.280262
12	29.8	159.2303	153.9603	159.2028	156.8912	157.2473	159.5876	155.3128	160.4259	158.7847	160.4468	156.256802	32.60200	26.047913
13	29.8	155.3653	160.8389	155.6822	159.451	159.5207	157.3406	160.4165	157.3768	155.0855	157.446	159.570083	29.28872	22.971229
14	16.5	173.1396	169.727	167.9631	173.8594	170.4103	165.759	165.2485	170.2899	170.2685	173.8222	170.499474	18.35933	12.822291
15	24	161.1813	161.1261	161.0415	161.5986	161.236	161.0932	161.0424	161.3521	160.7548	161.535	161.239301	27.61950	21.421208

[a] Averaged according to the Boltzmann-calculated contribution at the B3LYP/6–31+G (d,p) level.

[b] δ_U = Calculated Shielding Value (TMS) – Calculated Shielding Value (Averaged).

[c] $\delta_S = (\delta_U - 0.4551)/1.0769$.

Table S9. Experimental ^{1}H-NMR chemical shifts of compound **2** and GIAO isotropic magnetic shielding values calculated for the PCM/mPW1PW91/6–311+G (d,p) geometries of (6*S*,7*S*)/(6*R*,7*R*)-bisabol-2-en-11-ol-1,9-dione.

NO.	Expt-2	C1	C2	C3	C4	C5	C6	C7	C8	C9	C10	Averaged[9]
**1**	5.73	25.6731	25.5355	25.6123	25.8051	25.5552	25.774	25.6288	25.6041	25.5773	25.7733	25.5972348
**3a**	2.35	29.3634	29.3462	29.341	29.3192	29.3797	29.4678	29.3816	29.3839	29.5225	29.3422	29.3560408
**3b**	2.35	29.485	29.4231	29.4365	29.5092	29.4657	29.4485	29.3495	29.4661	29.2008	29.5248	29.4483945
**4a**	1.74	30.1149	29.8491	29.7992	30.0806	29.867	30.0097	29.9656	29.8968	29.6398	30.0721	29.8995152
**4b**	1.96	29.7847	30.3119	30.2952	29.8239	30.3764	29.5609	29.375	30.3531	29.8418	29.8339	30.2195544
**5**	2.15	29.0791	29.4844	29.5747	29.2388	29.5359	29.8295	29.7267	29.5844	29.4882	29.5356	29.4866166
**7a**	2.57	29.6123	28.2174	29.6601	29.6999	29.5993	28.4835	28.6912	28.6131	29.602	28.956	28.6555603
**7b**	2.57	28.8951	29.7156	28.9951	28.9636	28.1227	29.4029	29.7211	29.3494	28.9313	29.5164	29.3905212
**9a**	2.46	27.7475	28.9356	29.0458	27.5938	29.5394	29.7822	29.7636	29.4929	28.4562	27.8257	28.8713101
**9b**	2.58	29.3958	30.0853	30.0106	29.2676	30.1371	29.4047	29.1752	29.9727	29.6332	29.4738	29.9264784
**10**	2.79	29.8783	28.9062	29.2068	29.8966	28.9936	28.525	28.6901	28.767	29.4828	29.8279	29.0656003
**12**	1.19	30.8238	30.473	30.5161	30.628	30.5953	30.8852	30.5993	30.7344	30.5764	30.7365	30.5706206
**12**	1.19	31.3303	30.4157	30.4668	30.6671	30.6165	30.4835	30.5293	30.4919	30.784	30.4278	30.4887882
**12**	1.19	30.761	30.569	30.5896	30.7332	30.6559	30.6408	30.7885	30.8685	30.6174	30.8955	30.6651565
**13**	1.2	30.6279	30.7173	30.594	30.8058	30.6508	30.7248	30.8038	30.6236	30.5592	30.682	30.6988198
**13**	1.2	30.4452	30.9365	30.5012	31.1913	30.5735	30.8044	30.1362	30.6293	30.4264	30.725	30.8452205
**13**	1.2	30.8294	30.8554	30.7755	30.7991	30.6577	30.8072	30.9044	30.7272	30.7644	30.851	30.8085834
**14**	0.79	30.6621	31.0896	31.0167	30.8313	31.1118	30.8753	30.9432	31.0558	30.6407	30.8612	31.0319349
**14**	0.79	31.1816	30.8597	30.2908	31.2328	30.741	30.756	30.7971	30.944	31.0672	31.2453	30.9190909
**14**	0.79	31.0565	30.9103	30.9631	30.6957	30.9027	30.7159	30.7285	30.9002	31.2264	30.7174	30.8761825
**15**	1.92	29.8246	29.8156	29.8215	29.8784	29.816	29.8505	29.7805	29.8345	29.7751	29.8734	29.828866
**15**	1.92	29.692	29.6777	29.6667	29.7663	29.733	29.685	29.5919	29.7365	29.7984	29.7359	29.7052643
**15**	1.92	25.6731	25.5355	25.6123	25.8051	25.5552	25.774	25.6288	25.6041	25.5773	25.7733	29.7948757

[a] Averaged according to the Boltzmann-calculated contribution at the B3LYP/6–31+G (d,p) level.

TableS10. Experimental ^{13}C-NMR chemical shifts of compound **2** and GIAO isotropic magnetic shielding values calculated for PCM/mPW1PW91/6–311+G (d,p) geometries of (6*S*,7*R*)/(6*R*,7*S*)-bisabol-2-en-11-ol-1,9-dione.

NO	Expt-2	C1	C2	C3	C4	C5	C6	C7	C8	Averaged[a]	Unscaled shifts (δ_U)[b]	Scaled shifts (δ_S)[c]
**1**	127.2	57.3649	56.3223	55.3283	55.9175	55.7725	56.375	57.0258	55.3046	56.4451763	132.4136237	122.8628975
**2**	162.4	6.793	8.7647	7.166	7.4958	7.9054	10.9984	10.0705	11.0427	9.371124	179.487676	166.8943747
**3**	31.1	158.0289	157.9436	153.4627	152.9955	152.7507	157.9903	158.3623	153.1142	157.647426	31.2113736	28.20173379
**4**	23.8	159.9795	159.2796	157.6416	158.0158	157.8027	160.019	160.6429	156.7999	159.517534	29.3412659	26.45249827
**5**	50.2	139.7507	132.2417	139.7688	134.8381	133.9449	132.1158	138.4577	138.7231	133.568907	55.2898926	50.72396651
**6**	199.9	-27.9846	-27.7856	-26.8938	-26.4957	-26.2177	-25.4816	-25.8186	-23.0533	-26.742434	215.6012341	200.6737762
**7**	55	128.1135	129.9107	127.4123	128.1899	132.7298	132.4755	131.6449	133.6223	130.598375	58.2604252	53.50250229
**8**	211.5	-37.8394	-35.3133	-38.9939	-38.8589	-39.1688	-38.6652	-41.4229	-40.8296	-37.390045	226.2488448	210.6331913
**9**	49.8	137.1837	131.5333	135.0556	131.9401	129.7951	136.1903	139.8898	138.7383	134.433951	54.424849	49.91483397
**10**	27.3	154.5545	158.7769	153.1208	157.3721	153.8673	156.1269	154.7858	152.9894	156.691724	32.1670764	29.09566589
**11**	69.9	113.6082	113.9332	113.8921	113.4789	113.8749	115.2836	115.1914	114.7211	114.350794	74.5080061	68.69994023
**12**	29.8	155.5366	160.4241	155.5378	159.2323	153.7953	157.5049	159.6427	159.6492	158.343653	30.5151474	27.55050734
**13**	29.8	159.7437	155.3684	159.5449	155.6733	160.6707	159.2074	157.3302	157.24	157.447467	31.4113332	28.38876925
**14**	16.5	168.0626	166.5024	166.2217	167.6177	170.0482	166.9302	167.1703	165.1678	166.801528	22.0572725	19.63929707
**15**	24	161.138	161.1711	161.3803	161.432	161.4883	161.3015	161.3085	161.7409	161.07391	27.78489	24.99671686

[a] Averaged according to the Boltzmann-calculated contribution at the B3LYP/6–31+G (d,p) level.

[b] δ_U = Calculated Shielding Value (TMS) – Calculated Shielding Value (Averaged).

[c] $\delta_S = (\delta_U - 0.4551)/1.0769$.

Table S11. Experimental ^{1}H-NMR chemical shifts of compound **2** and GIAO isotropic magnetic shielding values calculated for PCM/mPW1PW91/6–311+G (d,p) geometries of (6*S*,7*R*)/(6*R*,7*S*)-bisabol-2-en-11-ol-1,9-dione.

NO	Expt-2	C1	C2	C3	C4	C5	C6	C7	C8	Averaged[a]
**1**	5.73	25.7618	25.7836	25.7271	25.6921	25.6044	25.9098	25.7853	25.8192	25.7982767
**3a**	2.35	29.6846	29.6854	29.362	29.3461	29.317	29.7102	29.7117	29.3573	29.6530775
**3b**	2.35	29.3581	29.2505	29.484	29.468	29.4386	29.2702	29.3792	29.51	29.2591376
**4a**	1.74	29.8812	29.9463	29.6267	29.7406	29.7301	29.969	29.9092	29.5683	29.904779
**4b**	1.96	29.6933	29.7234	30.3437	30.3681	30.2781	29.7327	29.7291	30.3556	29.7174904
**5**	2.15	29.1543	30.1833	28.87	29.7458	29.6956	30.1715	29.4841	29.1869	29.9451962
**7a**	2.57	29.7212	29.0433	29.6847	28.9515	29.7522	28.9792	29.2699	29.3122	29.1045506
**7b**	2.57	29.0321	29.8126	28.9436	29.5999	28.1286	29.3238	29.0171	28.6368	29.4168118
**9a**	2.46	28.7781	29.5184	27.252	29.7524	30.0377	29.8786	29.3371	28.0057	29.5082059
**9b**	2.58	29.454	28.5753	29.4413	28.5315	28.5669	29.0339	29.7115	29.6645	28.911281
**10**	2.79	30.0809	29.3178	30.2113	29.8163	29.7228	29.0951	29.9799	30.4155	29.3688315
**12**	1.19	30.5707	30.7499	30.6203	30.5277	30.4308	30.7045	30.6956	30.8697	30.6664871
**12**	1.19	30.519	30.2138	30.4663	30.4644	30.407	30.8016	29.949	30.5248	30.4377242
**12**	1.19	30.7836	30.8627	30.833	30.5816	30.5587	30.8291	30.6217	30.9034	30.7849532
**13**	1.2	30.5883	30.5661	30.8394	30.5515	30.6645	30.786	30.6603	30.6921	30.6293774
**13**	1.2	30.3522	30.5607	31.2229	30.483	30.7776	30.2398	30.7803	30.7584	30.4006674
**13**	1.2	30.6388	30.7759	30.7841	30.7493	30.8236	30.7538	30.8067	30.8009	30.720573
**14**	0.79	30.5594	30.4456	30.6293	30.8373	30.9489	30.3723	30.4547	30.4956	30.4179299
**14**	0.79	30.9111	31.0146	31.121	30.7466	31.0354	30.9729	30.7776	31.0012	30.9352058
**14**	0.79	30.0273	31.021	30.3423	31.2766	31.2847	31.1163	30.0599	30.2731	30.837372
**15**	1.92	29.8124	29.741	29.8266	29.8262	29.7952	29.8259	29.8415	29.8709	29.7617968
**15**	1.92	29.7899	29.7093	29.6924	29.6867	29.6785	29.7399	29.8262	29.7422	29.70809
**15**	1.92	29.6787	29.6483	29.7672	29.7945	29.7988	29.6923	29.7044	29.8274	29.6482681

[a] Averaged according to the Boltzmann-calculated contribution at the B3LYP/6–31+G (d,p) level.

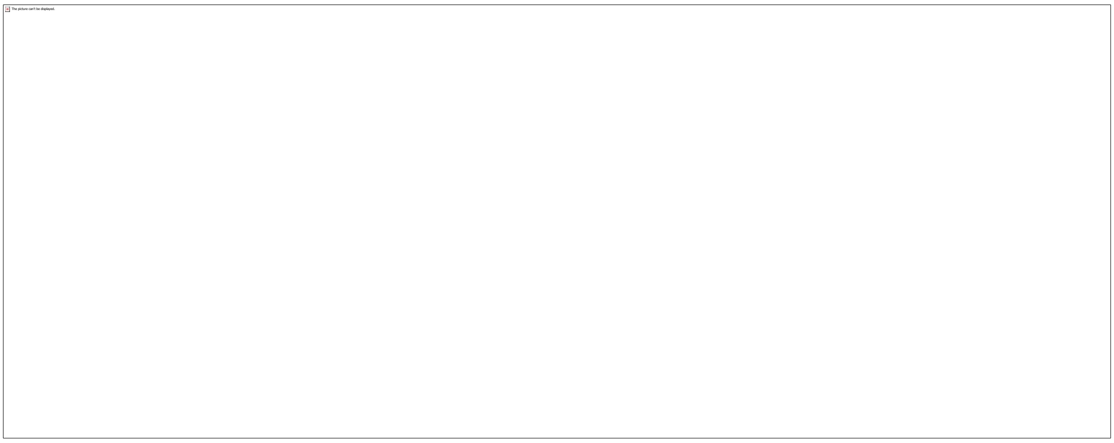

Figure S6. Regression analysis of the experimental ^{13}C NMR chemical shifts of compound **2** versus the calculated ^{13}C NMR chemical shifts of (6*S*,7*S*)/(6*R*,7*R*)-bisabol-2-en-11-ol-1,9-dione (A) and (6*S*,7*R*)/(6*R*,7*S*)-bisabol-2-en-11-ol-1,9-dione (B).

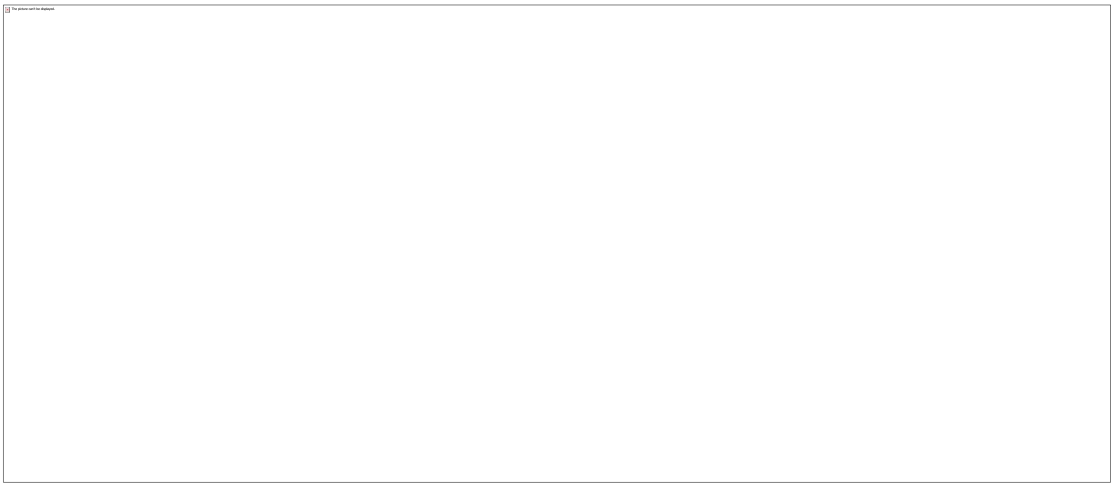

Figure S7. The calculated shielding tensors, scaled shifts, and unscaled shifts for (6*S*,7*S*)/(6*R*,7*R*)-bisabol-2-en-11-ol-1,9-dione (isomer 1) and (6*S*,7*R*)/(6*R*,7*S*)-bisabol-2-en-11-ol-1,9-dione (isomer 2).

Table S12. DP4+ probability (%) details of (6*S*,7*S*)/(6*R*,7*R*)-bisabol-2-en-11-ol-1,9-dione and (6*S*,7*R*)/(6*R*,7*S*)-bisabol-2-en-11-ol-1,9-dione for compound **2**.

Level of Theory: mPW1PW91/6–311+G (d,p) (acetone-*d_6*, PCM)

		(6*S*,7*S*)/(6*R*,7*R*)-bisabol-2-en-11-ol-1,9-dione	(6*S*,7*R*)/(6*R*,7*S*)-bisabol-2-en-11-ol-1,9-dione
sDP4+	^{1}*H*-date	80.02	19.98
	^{13}*C*-date	0	100
	All-date	0	100

uDP4+	** ^{1}*H*-date	50.13	49.87
	^{13}*C*-date	7.3	92.7
	All-date	7.34	92.66
DP4+	^{1}*H*-date	99.35	0.65
	^{13}*C*-date	1.08	98.92
	All-date	62.49	37.51

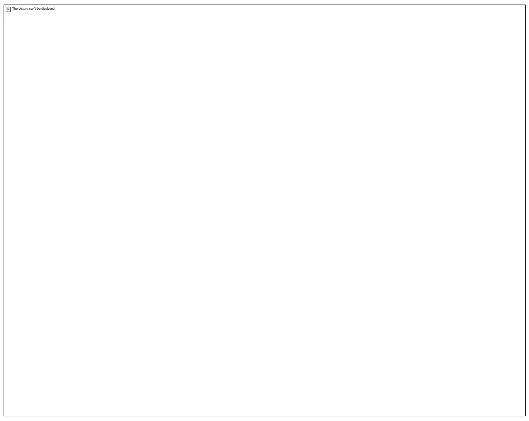

Figure S8. DP4+ probability of (6*S*,7*S*)/(6*R*,7*R*)-bisabol-2-en-11-ol-1,9-dione and (6*S*,7*R*)/(6*R*,7*S*)-bisabol-2-en-11-ol-1,9-dione for compound **2**.

Text S3. ECD Calculation of Compound 1.

Conformation searches and DFT optimization at the B3LYP/6-31G (d) level of (6*S*,7*S*)-bisabol-2-en-11-ol-1,9-dione are the same as described in section 1. The B3LYP/6–31G (d)-optimized 10 conformers were then reoptimized at the *ω*B97XD/DGDZVP level in acetonitrile. ECD computations for the *ω*B97XD/DGDZVP-optimized conformers were carried out at the CAM-B3LYP/DGDZVP level in acetonitrile [3]. According to the Boltzmann distribution theory and their relative Gibbs free energy (ΔG), the calculated ECD spectrum for (6*S*,7*S*)-bisabol-2-en-11-ol-1,9-dione was generated using SpecDis 1.71 with σ = 0.25 eV

and a UV shift of +10 nm [5]. The corresponding theoretical ECD spectrum of (6*R*,7*R*)-bisabol-2-en-11-ol-1,9-dione was depicted by inverting that of (6*S*,7*S*)-bisabol-2-en-11-ol-1,9-dione. In the region of 190–350 nm, the theoretically calculated ECD spectrum of (6*S*,7*S*)-bisabol-2-en-11-ol-1,9-dione matched with the experimental ECD spectrum of compound **1** (Figure S9).

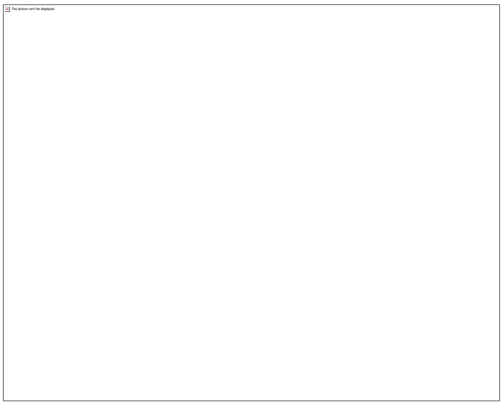

Figure S9. Experimental and calculated ECD spectra of compound **1**.

Text S4. ECD Calculation of Compound 2.

Conformation searches and DFT optimization at the B3LYP/6-31G (d) level of (6*S*,7*R*)-bisabol-2-en-11-ol-1,9-dione are the same as described in section 1. The B3LYP/6–31G(d)-optimized eight conformers were then reoptimized at the *ω*B97XD/DGDZVP level in acetonitrile. ECD computations for the *ω*B97XD/DGDZVP-optimized conformers were carried out at the CAM–B3LYP/DGDZVP level in acetonitrile [3]. According to the Boltzmann distribution theory and their relative Gibbs free energy (ΔG), the calculated ECD spectrum for (6*S*,7*R*)-bisabol-2-en-11-ol-1,9-dione was generated using SpecDis 1.71 with σ = 0.3 eV and

a UV shift of −10 nm [5]. The corresponding theoretical ECD spectrum of (6*R*,7*S*)-bisabol-2-en-11-ol-1,9-dione was depicted by inverting that of (6*S*,7*R*)-bisabol-2-en-11-ol-1,9-dione. In the region of 190–360 nm, the theoretically calculated ECD spectrum of (6*R*,7*S*)-bisabol-2-en-11-ol-1,9-dione matched with the experimental ECD spectrum of compound **2** (Figure S10).

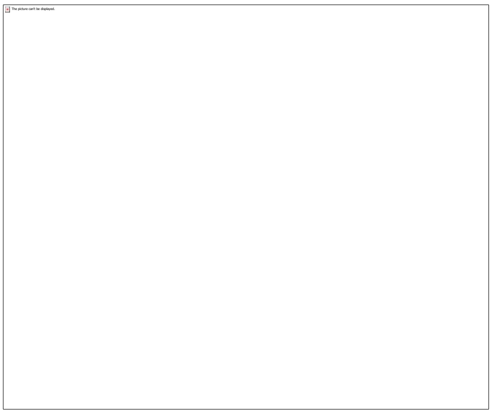

Figure S10. Experimental and calculated ECD spectra of compound **2**.

Text S5. ECD Calculation of Compound 3.

Conformation searches based on molecular mechanics with MMFF94s force field were performed for (6*S*,7*S*)-bisabol-2,10-dien-1,9-dione and (6*S*,7*R*)-bisabol-2,10-dien-1,9-dione and gave seven and 15 conformers having relative energies within 3.5 kcal/mol, respectively [1]. The conformers were optimized using DFT at the B3LYP/6–31G (d) level in vacuum with the

Gaussian 16 program (Tables S13 and S14).[2] The B3LYP/6–31G (d)-optimized conformers were then reoptimized at the *ω*B97XD/DGDZVP level in acetonitrile. ECD computations for the *ω*B97XD/DGDZVP-optimized conformers (Figures S11 and S12) were carried out at the CAM–B3LYP/DGDZVP level in acetonitrile [3]. According to the Boltzmann distribution theory and their relative Gibbs free energy (ΔG), the calculated ECD spectrum for (6*S*,7*S*)-bisabol-2,10-dien-1,9-dione was generated using SpecDis 1.71 with σ = 0.25 eV and a UV shift of +10 nm, while the spectrum for (6*S*,7*R*)-bisabol-2,10-dien-1,9-dione was generated using SpecDis 1.71 with σ = 0.3 eV and a UV shift of +10 nm [5]. In the region of 210–360 nm, the theoretically calculated ECD spectrum of (6*S*,7*S*)-bisabol-2,10-dien-1,9-dione matched with the experimental ECD spectrum of compound **3** (Figure S13).

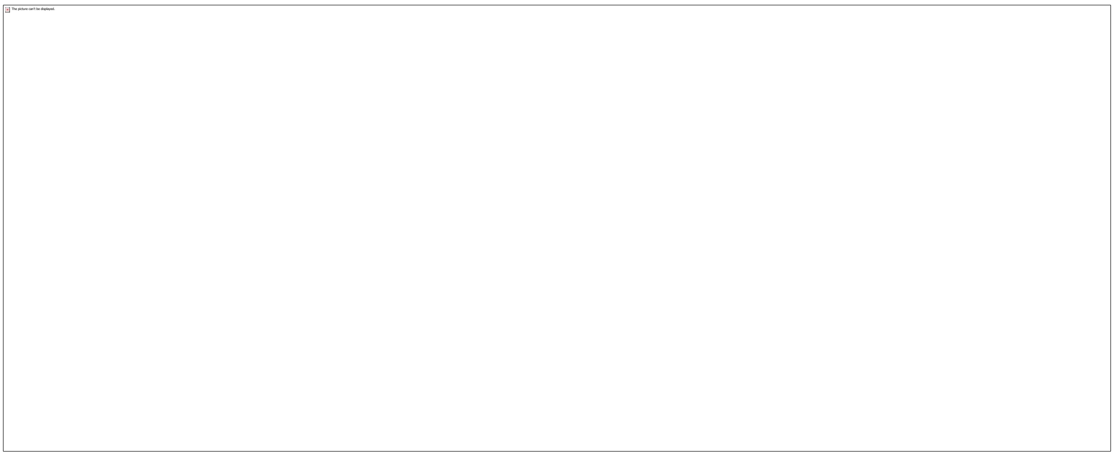

Figure S11. *ω*B97XD/DGDZVP optimized seven conformers of (6*S*,7*S*)-bisabol-2,10-dien-1,9-dione.

Table S13. Energy analysis for the conformers of (6*S*,7*S*)-bisabol-2,10-dien-1,9-dione.

Conf.	MMFF energy	BL3YP/6-31G(d) Gibbs free energy (298.15 K)			ωB97XD/DGDZVP Gibbs free energy (298.15 K)		
	ΔE (Kcal/mol)	G (Hartree)	ΔG (Kcal/mol)	Boltzmann distribution	G (Hartree)	ΔG (Kcal/mol)	Boltzmann distribution
C1	0.0000	-734.996	0	0.494	-734.587	0	0.239
C2	0.2815	-734.995	0.565	0.19	-734.587	0.457	0.11
C3	0.3983	-734.995	0.867	0.114	-734.588	-0.438	0.5
C4	0.5609	-734.995	0.564	0.191	-734.587	0.457	0.11
C5	1.0842	-734.99	4.058	0.001	-734.582	3.609	0.001
C6	1.3027	-734.989	4.631	0	-734.581	4.054	0
C7	1.6045	-734.992	2.267	0.011	-734.586	1.062	0.04

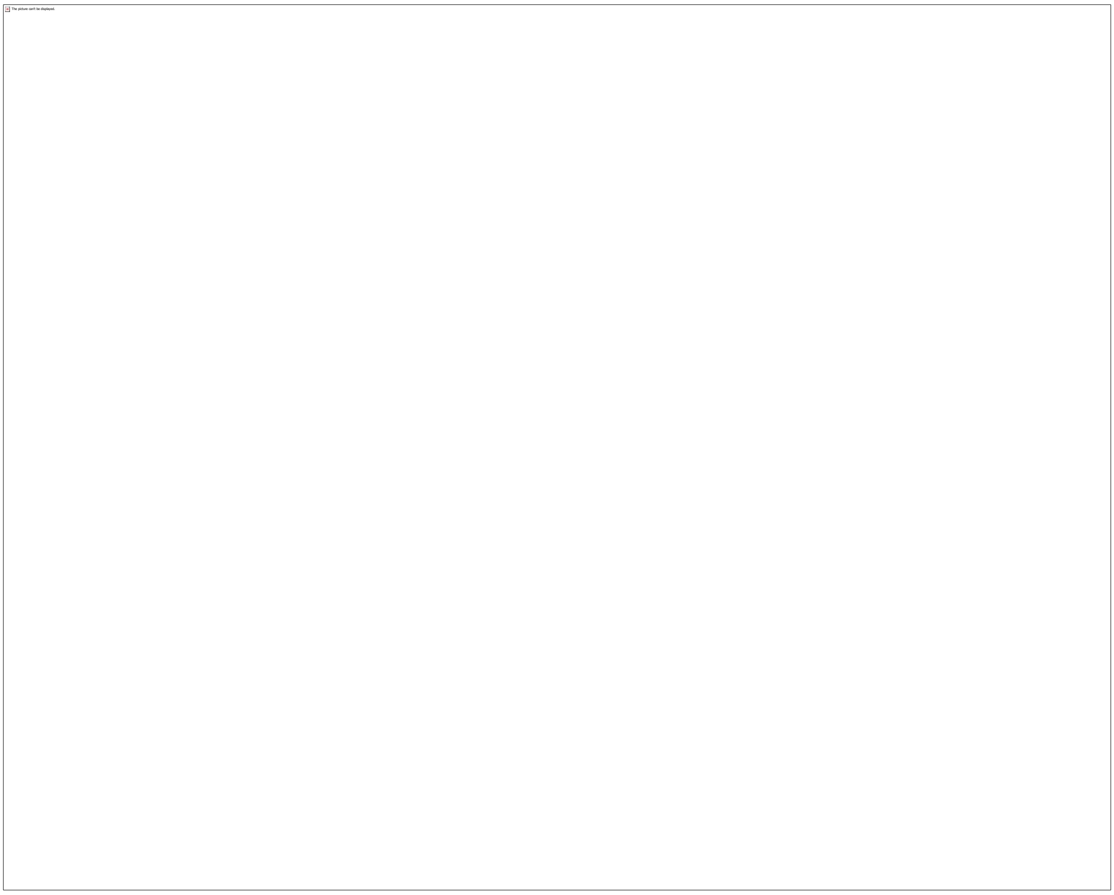

Figure S12. *ω*B97XD/DGDZVP optimized 15 conformers of (6*S*,7*R*)-bisabol-2,10-dien-1,9-dione.

Table S14. Energy analysis for the conformers of (6*S*,7*R*)-bisabol-2,10-dien-1,9-dione.

Conf.	MMFF energy	BL3YP/6-31G(d) Gibbs free energy (298.15 K)			ωB97XD/DGDZVP Gibbs free energy (298.15 K)		
	ΔE (Kcal/mol)	G (Hartree)	ΔG (Kcal/mol)	Boltzmann distribution	G (Hartree)	ΔG (Kcal/mol)	Boltzmann distribution
C1	0.0000	-734.995	0	0.224	-734.586201	0	0.033
C2	0.0171	-734.993	0.963	0.044	-734.588354	-1.351	0.326
C3	0.1208	-734.993	0.93	0.047	-734.586202	-0.001	0.033
C4	0.1900	-734.995	0.005	0.222	-734.583304	1.818	0.002
C5	0.2869	-734.992	1.393	0.021	-734.585342	0.539	0.013
C6	0.3028	-734.993	1.291	0.025	-734.585105	0.688	0.01
C7	0.3103	-734.993	0.811	0.057	-734.58399	1.387	0.003
C8	0.3312	-734.991	2.192	0.006	-734.585209	0.622	0.012
C9	0.3473	-734.988	4.195	0	-734.581228	3.121	0
C10	0.3689	-734.99	3.144	0.001	-734.585436	0.48	0.015
C11	0.4866	-734.994	0.737	0.064	-734.588073	-1.175	0.242
C12	0.5874	-734.994	0.134	0.178	-734.587549	-0.846	0.139
C13	0.6612	-734.993	0.824	0.056	-734.58708	-0.552	0.085
C14	0.8962	-734.993	0.826	0.055	-734.587093	-0.56	0.086
C15	1.0512	-734.985	6.031	0	-734.577905	5.206	0

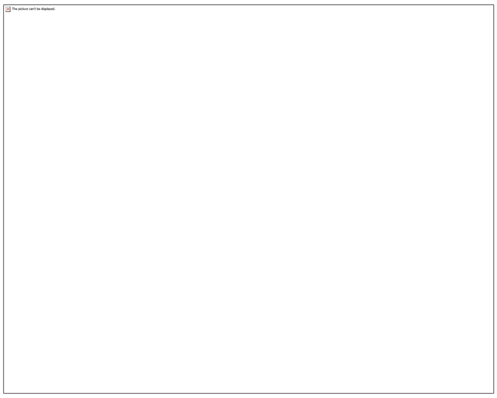

Figure S13. Experimental and calculated ECD spectra of compound **3**.

Text S6. ECD Calculation of Compound 4.

The ECD calculations of (6*S*,7*S*)-bisabol-2,10-dien-1,9-dione and (6*S*,7*R*)-bisabol-2,10-dien-1,9-dione are the same as described in section 5. The corresponding theoretical ECD spectra of (6*R*,7*R*)-bisabol-2,10-dien-1,9-dione and (6*R*,7*S*)-bisabol-2,10-dien-1,9-dione were depicted by inverting those of (6*S*,7*S*)-bisabol-2,10-dien-1,9-dione and (6*S*,7*R*)-bisabol-2,10-dien-1,9-dione, respectively. In the region of 200–360 nm, the theoretically calculated ECD spectrum of (6*R*,7*S*)-bisabol-2,10-dien-1,9-dione matched with the experimental ECD spectrum of compound **4** (Figure S14).

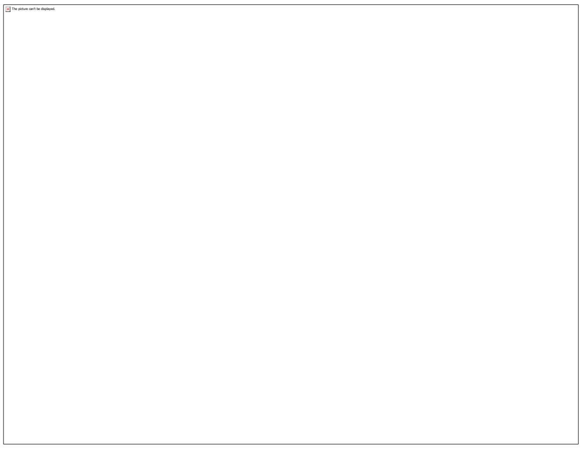

Figure S14. Experimental and calculated ECD spectra of compound **4**.

7. Original Spectra of New Compounds

The picture can't be displayed.

Figure S15. The UV spectrum of compound **1** in MeCN.

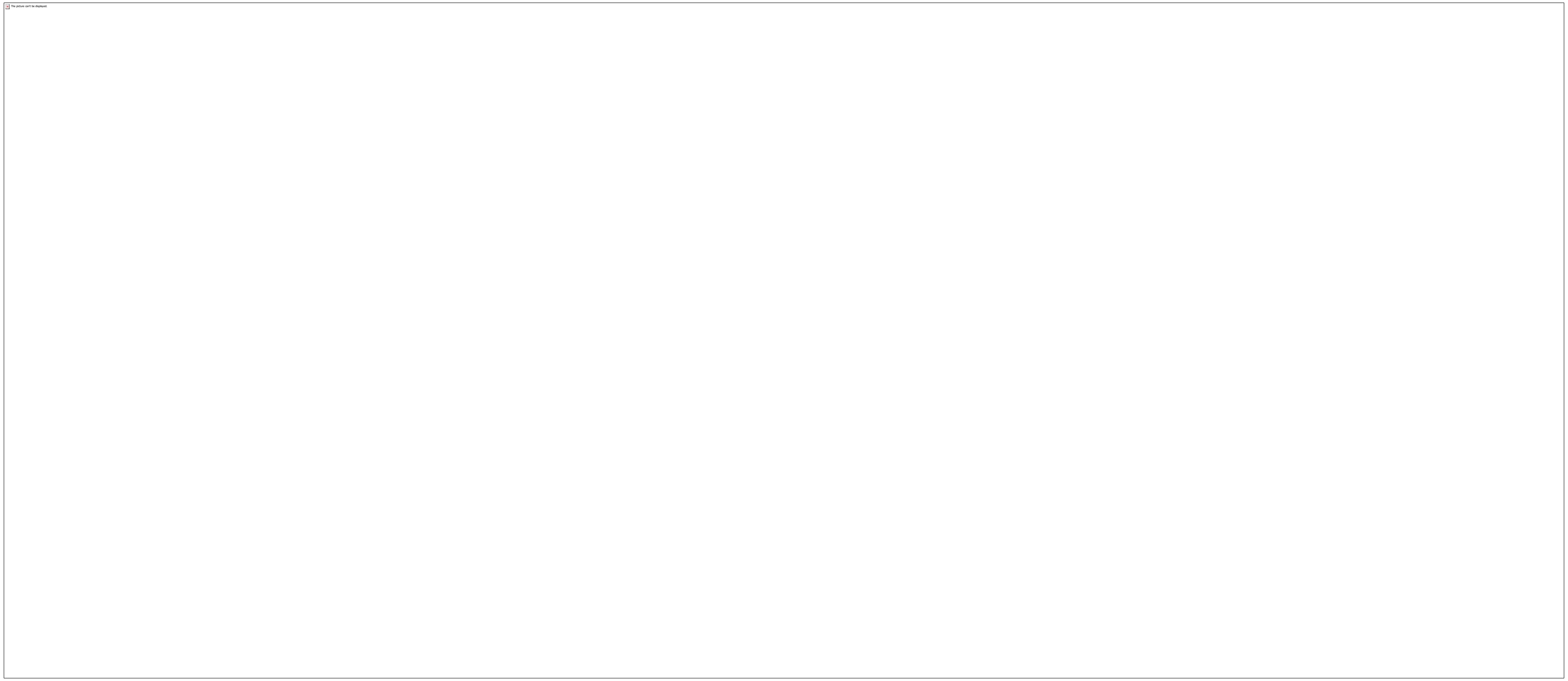

Figure S16. The IR spectrum of compound **1**.

Figure S17. The (+)-HRESIMS spectroscopic data of compound **1**.

Figure S18. The ^{1}H NMR spectrum of compound **1** in acetone-d_6.

Figure S19. The ^{13}C NMR spectrum of compound **1** in acetone-d_6.

Figure S20. The DEPT spectrum of compound **1** in acetone-d_6.

Figure S21. The HSQC spectrum of compound **1** in acetone-d_6.

Figure S22. The ^{1}H-^{1}H COSY spectrum of compound **1** in acetone-d_6.

Figure S23. The HMBC spectrum of compound **1** in acetone-d_6.

The picture can't be displayed.

Figure S24. The UV spectrum of compound **2** in MeCN.

The picture can't be displayed.

Figure S25. The IR spectrum of compound **2**.

The picture can't be displayed.

Figure S26. The (+)-HRESIMS spectroscopic data of compound **2.**

Figure S27. The ^{1}H NMR spectrum of compound **2** in acetone-d_6.

Figure S28. The ^{13}C NMR spectrum of compound **2** in acetone-d_6.

Figure S29. The DEPT spectrum of compound **2** in acetone-d_6.

Figure S30. The HSQC spectrum of compound **2** in acetone-d_6.

Figure S31. The ^1H-^1H COSY spectrum of compound **2** in acetone-d_6.

Figure S32. The HMBC spectrum of compound **2** in acetone-d_6.

The picture can't be displayed.

Figure S33. The UV spectrum of compound **3** in MeCN.

The picture can't be displayed.

Figure S34. The IR spectrum of compound **3**.

The picture can't be displayed.

Figure S35. The (+)-HRESIMS spectroscopic data of compound **3**.

Figure S36. The ^{1}H NMR spectrum of compound **3** in $CDCl_3$.

Figure S37. The ^{13}C NMR spectrum of compound **3** in $CDCl_3$.

Figure S38. The DEPT spectrum of compound **3** in $CDCl_3$.

Figure S39. The HSQC spectrum of compound **3** in $CDCl_3$.

Figure S40. The ^1H-^1H COSY spectrum of compound **3** in $CDCl_3$.

Figure S41. The HMBC spectrum of compound **3** in $CDCl_3$.

The picture can't be displayed.

Figure S42. The UV spectrum of compound **4**.

The picture can't be displayed.

Figure S43. The IR spectrum of compound **4**.

The picture can't be displayed.

Figure S44. The (+)-HRESIMS spectroscopic data of compound **4**.

Figure S45. The ^{1}H NMR spectrum of compound **4** in $CDCl_3$.

Figure S46. The ^{13}C NMR spectrum of compound **4** in $CDCl_3$.

Figure S47. The DEPT spectrum of compound **4** in $CDCl_3$.

Figure S48. The HSQC spectrum of compound **4** in $CDCl_3$.

Figure S49. The ^{1}H-^{1}H COSY spectrum of compound **4** in $CDCl_3$.

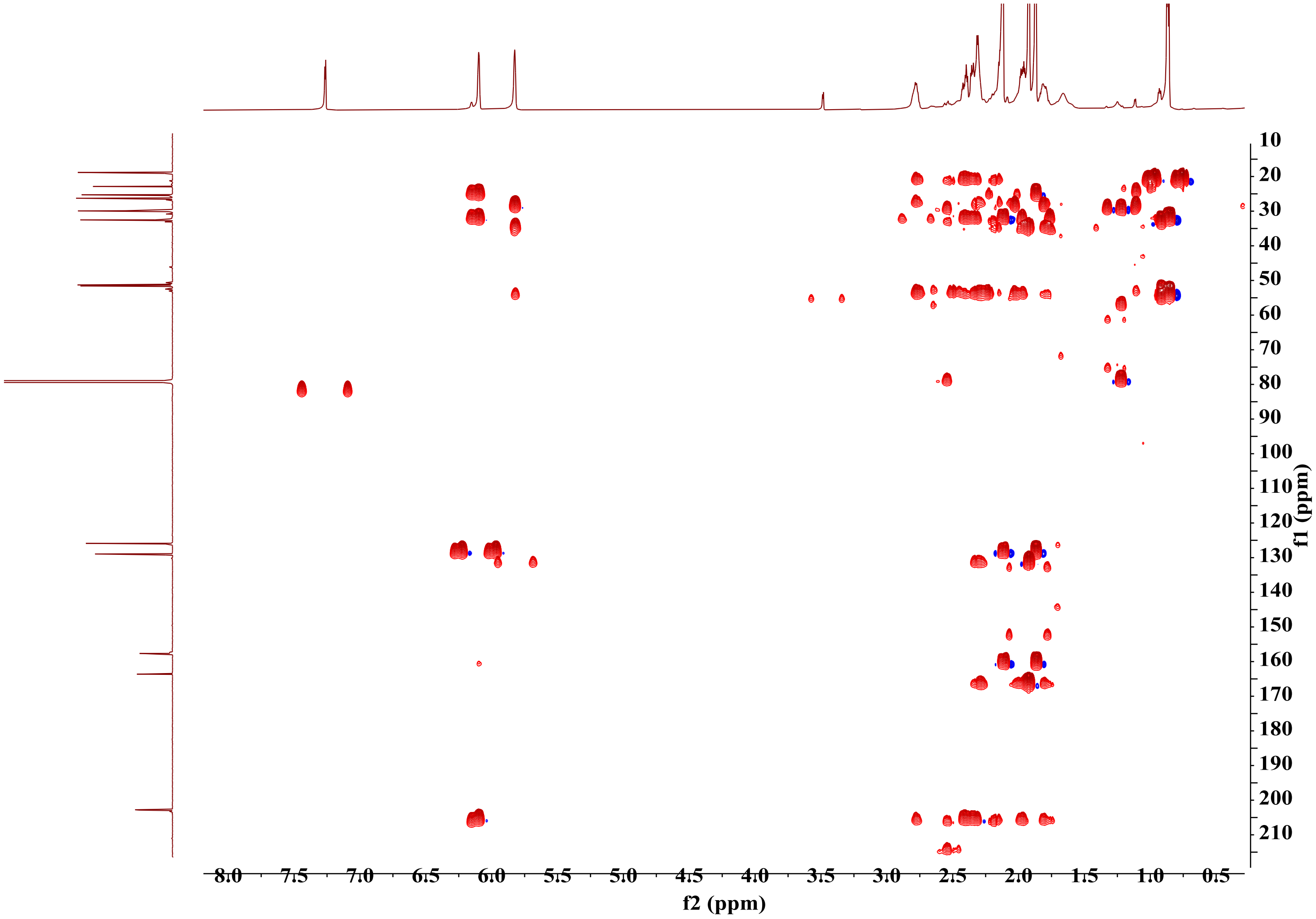

10
20
30
40
50
60
70
80
90
100
110
120
130
140
150
160
170
180
190
200
210
f1 (ppm)
8.0
7.5
7.0
6.5
6.0
5.5
5.0
4.5
4.0
3.5
3.0
2.5
2.0
1.5
1.0
0.5
f2 (ppm)

Figure S50. The HMBC spectrum of compound **4** in $CDCl_3$.

References:

Goto, H.; Osawa, E. Corner flapping: a simple and fast algorithm for exhaustive generation of ring conformations. *J Am Chem Soc.* **1989**, *111*, 8950−8951. https://doi.org/10.1021/ja00206a046.

Goto, H.; Osawa, E. An efficient algorithm for searching low-energy conformers of cyclic and acyclic molecules. *J Chem Soc, Perkin Trans 2.* **1993**, *2*, 187−198. https://doi.org/10.1039/P29930000187.

Frisch, M. J.; Trucks, G. W.; Schlegel, H. B.; Scuseria, G. E.; Robb, M. A.; Cheeseman, J. R.; Scalmani, G.; Barone, V.; Petersson, G. A.; Nakatsuji, H. et al. *Gaussian 16*, Revision C.01; Gaussian, Inc., Wallingford CT, **2016**.

Grimblat, N.; Zanardi, M. M.; Sarotti, A. M. Beyond DP4: an improved probability for the stereochemical assignment of isomeric compounds using quantum chemical calculations of NMR Shifts. *J Org Chem.* **2015**, *80*, 12526−12534. https://doi.org/10.1021/acs.joc.5b02396.

Gausssum 2.25: O'Boyle, N. M.; Tenderholt, A. L.; Langner, K. M. J. Cclib: a library for package-independent computational chemistry algorithms. *Comput Chem.* **2008**, *29*, 839–845. https://doi.org/10.1002/jcc.20823.
